# 

**Published:** 1834

**Authors:** 


### INDEX.

A.

Abdomen, Examination of, 509.
Actea Racemosa, 557.
Amaurosis, treatment of, 592.
Anatomy and Physiology, study of, 650.
Animal Diet, bad effects of, 566.
Animalcules in water, 155.
Annomalous disease, 182.
Anthelmintic Emulsion, 277.
Apocynum Canabinum, 146.
Atmospheric pressure, 116.

B.

Blood in Cholera, Pathology of, 428.
Blood-letting, local, 447.
Botany in Ohio, 476.
Bowels, inflation of, 602.
Bread, poisonous, 149.
Bronchitis, 282.
Broussais' doctrines, 417.

C.

Callous, formation of, 293.
Chloride of lime in itch, 594.
in Psora, 281.
in Burns, 283.
Cholera, 653. 557. 161. 43.
Ætiological remarks on, 163.
Exciting causes of, 168.
Treatment in Lexington, 169.
in Paris, 145.
Treated by cold, 133.
in Cincinnati, 172.
fatal cases of, 174.
in Yucatan, 321. 565.
in Columbus, 341.
Treatment, 347.
Cincinnati, 151.
Cincinnati Medical Society, 477.
Cold a cause of disease, 257.
Cold dash, 123. 292.
Contagion, 95.
Convulsions of infants, 123. 277.
caused by Cathartics, 155.
Copper in wheat, 611.
Croton oil, 549.
Cubebs a cause of an eruption, 273.
Cupping, 534.
Dry, 590. 605.

D.

Diet, excesses in, 228.
Dress, absurdities in, 230.
Dysmenorrhœa, 313.

E.

Editorial note, 363.
observations, 572.
Education, 226.
Education Convention, 486.
Electricity, animal, 263.
Electro-Galvanism, agency of, 613.
Electro-Therapeutics, 309.
Entomological cabinet, 319.
Epidemic constitution, 10.
in Cincinnati, 153.
Epidemics, Medical Statistics, and Political Economy, 431.
Ergot, 274. 275.
Euphorbia Corrolata, 147.
Exercise, 261.
neglect of, 233.

F.

Fatal disease at Stanford, Ky. 570.
Fever, Puerperal, 279.
Treatment, 300.
Scarlet, 10.
Varieties and stages, 16.
Pathology, 18.
Treatment, 19. 25.
Remarks on, 23.
Fingers, changes in, 272.
Foetus Acephalous, 568.
Food, quality of, 120.
Foramen Ovale open, 567.
Fracture of thigh and leg, 558.

G.

Gall Bladder, escape of contents, 518.
from softening of cystic duct, ib.
from rupture, ib.
from perforation, 521.
Galvanic Brush, 268.
Gangrene of the mouth, 295.
Gastritis, Pathology & treatment, 595.
Geology of Ohio, 356.
Gleet, remedy for, 566.
Gonorrhœa and treatment, 266.
Graduates, Western, 157.

Great Intestines, escape of contents, 514.

H.

Hæmorrhoids, excision of, 123,
Health of the West, 487.
Hemlock, 122.
Hermaphrodism, singular variety, 443.
Henbane, 122.
Hexade, second, 316.
Hospitals on Mississippi, 652.
Hydatids, conversion into tubercles, 593.
Hydrophobia prevented, 27.
Hysteralgia, 585.

I.

Ileum, perforation of, 511.
Immersion in confined atmosphere, 232.
Imponderable agents, 410.
Inflammation of bowels, 603.
Intestines, escape of contents, 510.
Iodine in Salivation, 602.
Infusora, functions of, 105.
  digestive system of, 107.
  muscular system of, 110.
  vascular system of, 114.
  nervous system of, 115.

J.

Jejunum, laceration of, 511.

K.

Kentucky, flora of, 319.
  Plants of, 474.

L.

Lectures, private, 157.
Lightning, effects of corrected, 456.
Lithotomy, 158.
Louisiana, diseases of, 187.
Lunatics in New-York, 617.

M.

Measles & Mumps in combination, 154.
Meat, poisonous changes in, 149.
Medical classes, 488.
Medical College of Ohio, 156. 623.
  appointments in, 320.
  Charges against Trustees, 635. 630.
    against Faculty, 629. 630.
  Dr. Staughton's letter, 634.
  Dr. Eberle's letters, 637.
  Evidence before Senate, 637.
  Report of Committee, 477.
  Remarks on Report, 645.
  Letters of Drs. Mitchell, Moorhead and Cobb, 647.
Medical Journal, new plan, 316.
Medical Law of Ohio repealed, 479.
Medical Obituary, 488.
Medicine, certainty of, 568.
Medicine and Surgery, Cyclopædia of, 385.
Meteorological Regester, 656.
Moorhead's paper, remarks on, 524.
Muscular contraction, 263.

N.

Nitrate of Silver, application of, 273. 283.

O.

Oil of Tansy, a poison, 569.
Ophthalmia, Indian, 450.
Opium in large doses, 139.
Obituary Notices, 156. 655.
Osteo Sarcoma, 451.
Ovarian extirpation, 436.

P.

Paralysis, of one side of face, 673.
Peritoneal iritation and inflammation, 489.
Periostosis, Dr. Graves on,
Physical Education, 226.
Physiology, 193.
Plants, specimens of, 659.
Portal, N. death of, 424.
Prevention of disease by cold, 259.
Professional duties extra, 316.
Prolapsis Ani, 284.
  treatment of, 131. 594.
Purgatives, 52.

R.

Rectum, perforation of, 515.
Reproduction of crystaline lens, 429.
Residence for consumptives, 487.
Rheumatism, spinal origin, 305.

S.

School for the blind, 483.
Sensibility of the skin, 439.
Serum of blood, 594.
Shooting stars, 368.
Sloughing & removal of Intestine, 320.
Sleep, 232.
Stimulants external, 135.
Stimulating drinks, abuse of, 229.
Stomach, perforation of, 508.
  fissure of pylorous, ib.
  ulceration of, 425.
Strichnine in paralysis, 532.
Sulphate of Quinine, 452.

T.

Tartor emetic in pneumonia, 144.
  in pulmonary inflammation, 265
Theory of sounds of the heart, 274.
Tobacco, abuse of, 230.
Tubercular disease, 653.
Turpentine in Sciatica, 592.
Tropical plants, 486.

U.

Uterine hæmorrhage, 437.
Urinary Caculi, removal of, 539.
Urine, morbid constitution of, 444.

V.

Vacination in free schools, 156.
Varices, cure of, 610.
Veins, admission of air into, 563.

W.

Water, as a remedial agent, 424.
Westren Reserve, topography of, 12.
Worms in the bladder, 354.

